# Targeting autophagy as a therapeutic strategy in pediatric acute lymphoblastic leukemia

**DOI:** 10.1038/s41598-024-54400-6

**Published:** 2024-02-18

**Authors:** Henri Colyn Bwanika, Isabelle Rose Leo, Nona Struyf, Asimina Talanti, Luay Aswad, Aishwarya Konnur, Ann-Charlotte Björklund, Mats Heyman, Georgios Rassidakis, Tom Erkers, Brinton Seashore-Ludlow, Rozbeh Jafari, Katja Pokrovskaja Tamm

**Affiliations:** 1 Department of Oncology and Pathology, Karolinska Institutet, Akademiska stråket 1, BioClinicum J6:14, 17164 Solna, Sweden; 2 Science for Life Laboratory, Solna, Sweden; 3 Center for Hematology and Regenerative Medicine, Department of Medicine, Karolinska Institute, Huddinge, Sweden; 4 Childhood Cancer Research Unit, Department of Women’s and Children’s Health, Karolinska Institutet, Stockholm, Sweden

**Keywords:** Cell biology, Drug discovery, Cancer, Cancer therapy, Haematological cancer, Paediatric cancer

## Abstract

Autophagy is activated in response to a variety of stress conditions including anti-cancer therapies, and tumors cells often depend on autophagy for survival. In this study, we have evaluated inhibition of autophagy as therapeutic strategy in acute lymphoblastic leukemia (ALL) in children, both as a single treatment and in combination with glucocorticoid (GC) Dexamethasone (Dexa). Analysis of proteomics and RNA-seq of ALL cell lines and primary samples identified an upregulation of Vps34 and ATG14 proteins and autophagy and lysosomal pathway enrichment in a genetic subgroup with a recurrent t(12;21) translocation. Cells from this sugbroup were also significantly more sensitive to the selective autophagy or lysosomal inhibitors than cells with other genetic rearrangements. Further, combination of Dexa with either lysosomal or autophagy inhibitors was either synergistic or additive in killing leukemic cells across various genetic and lineage backgrounds, for both cell lines and primary samples, as assessed using viability assays and SynergyFinder as well as apoptotic caspase 3/7-based live-cell assays. Our data demonstrate that targeting autophagy represents a promising strategy for the treatment of pediatric ALL, both as a selective modality for the t(12;21) pre-B-ALL subgroup, and in combination treatments to sensitize to GC-induced cytotoxicity.

## Introduction

Autophagy is a conserved cellular program utilized by the eukaryotic cell to recycle its own constituents, such as damaged organelles or protein aggregates. It is characterized by the formation of autophagosomes, double membrane-surrounded vesicles that engulf protein aggregates or organelles, like mitochondria, and then fuse with lysosomes where the cargo is degraded^1^. The lipid kinase Vps34, also known as class III phosphatidylinositol-3 kinase, plays a central role in autophagy initiation, which is manifested by a formation of a protein complex between Vps34, Vps15, Beclin-1 and ATG14, called PI3-kinase Complex I, leading to its tethering to a membrane and initiation of formation of an autophagosome. Vps34 is also involved in endosomal trafficking, and to perform this function forms another type of complex, referred to as Complex II^2,3^. Autophagy is activated as a survival strategy in response to stressors such as starvation, hypoxia, and cytotoxic anti-cancer therapy, as we as others have established^4,5^. Thus, the discovery that autophagy may limit the cytotoxic effects of anti-cancer drugs has led to the initiation of clinical trials using the lysosomal inhibitor chloroquine (CQ) or hydroxychloroquine (HCQ) in combination therapies^1^. We and others have also shown that combination of anti-cancer drugs with more selective autophagy inhibitors, such as Vps34 inhibitors, had synergistic or additive effects in breast cancer models^4^. Several companies have developed selective Vps34 inhibitors: SB02024 by Sprint Bioscience^4^, SAR405 by Sanofi^6^ and a more potent PIK-III by Novartis^7^. Another inhibitor targeting selectively PI3-kinase Complex I has been reported to disrupt complex formation between ATG14 and Beclin-1^8^.

Acute lymphoblastic leukemia (ALL) is the most common type of cancer in children^9^. There are more than ten different genetic subgroups in pediatric ALL characterized by recurring genetic abnormalities such as chromosomal translocations, gene amplifications and mutations^10^. The stratification of patients for treatment is based on genetic aberrations, age, and immune cell type, and treatment regimens typically involve similar drugs adjusted to risk stratification by changes in frequency and doseage. These drugs include high dose of the metabolic hormones glucocorticoids (GC), such as Dexamethasone (Dexa), in combination with L-asparaginase and multi-agent chemotherapy. Despite the high current survival rate of 90%, some 20% of pediatric ALL patients relapse owing to the development of resistance to therapy and many more are at risk for long-term toxicities^9^. Each of the genetic sub-groups with subtle genetic abnormalities may, however, present with unique opportunities for targeted therapy, and thus may benefit from a personalized treatment approach, such as recurring t(9;22) translocation in leukemic cells, where targeted therapy against BCR-ABL is applied. Interestingly, a recent study revealed that in a genetic sub-group of ALL with a recurrent t(12;21) resulting in ETV6-RUNX1 fusion protein (also known as TEL-AML1), Vps34 is up-regulated^11^. This study showed that such cells are particularly sensitive to the lysosomal inhibitor CQ.

GCs are essential drugs in the treatment of ALL, and general treatment failure has been related specifically to GC-resistance^12^. We have previously revealed a profound induction of autophagy in GC-treated cells and suggested its involvement in GC-induced cell death^13^. In a more recent study, applying proteomics and metabolomics, we found that lysosomal enzymes were also upregulated by Dexa^14^. The present study was designed to determine whether autophagy inhibition can be beneficial for the treatment of pediatric ALL. For this, we assessed expression levels of Vps34 and sensitivity to autophagy inhibition in leukemic cells with different genetic backgrounds and asked whether inhibition of autophagy will potentiate or protect cells from GC-induced toxicity. We used pharmacological inhibition of either the initial stage of autophagy or of the lysosomal stage alone or in combination with Dexa treatment, and both in the cell lines and primary ALL cells.

## Results

### Autophagy is elevated in leukemic cells with the t(12:21) translocation

**Figure 1:**
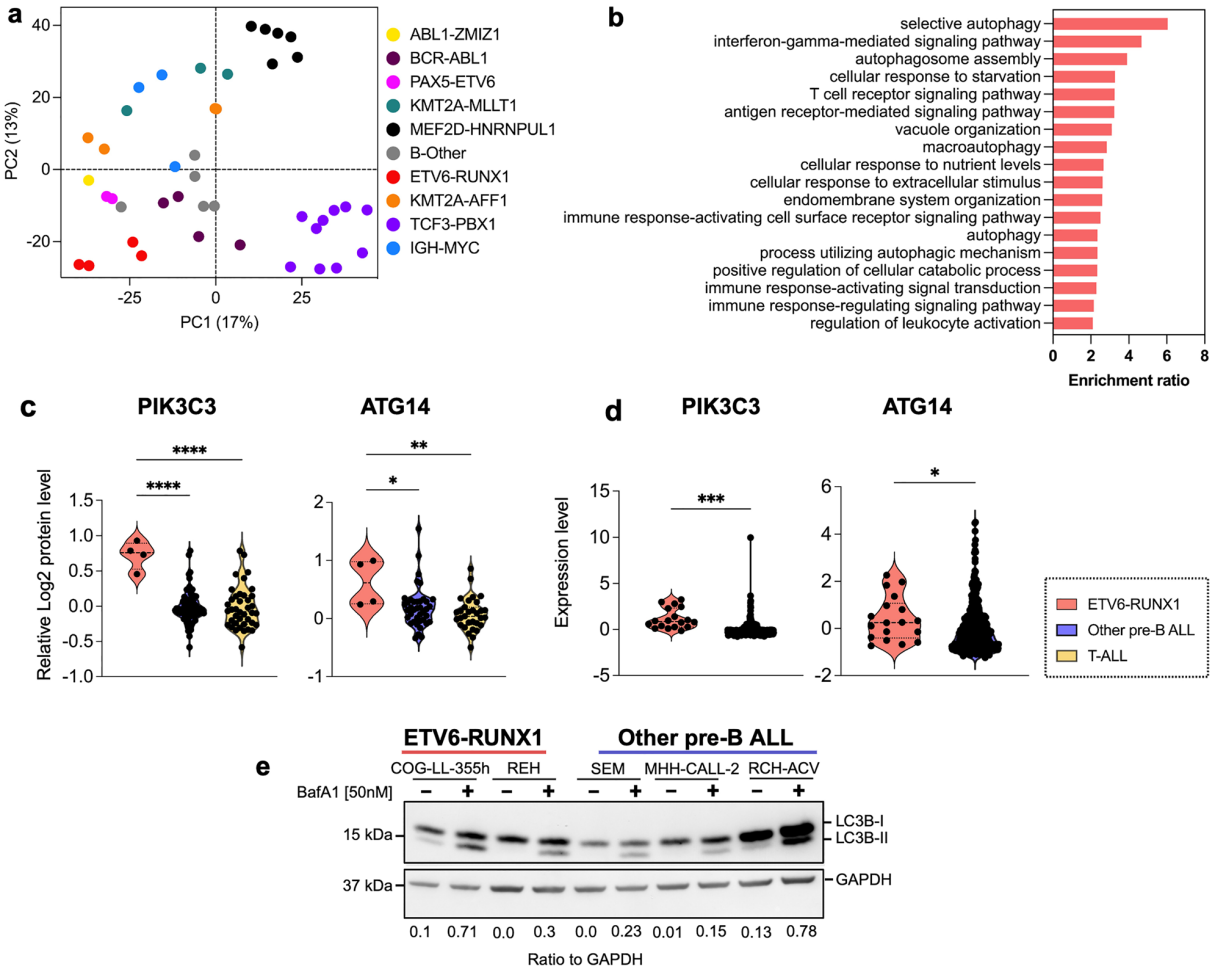
Activation of autophagy and lysosomal pathways in ALL cells with ETV6-RUNX1 translocation. (**a**) Clustering according to the cytogenetic background of 41 pre-B ALL cell lines by Principal component analysis (PCA) of highly variable genes from RNAseq data. (**b**) Gene ontology—Biological process analysis of up-regulated genes in ETV6-RUNX1 cell lines as compared to all the other ALL cell lines. (**c**) Expression levels of Vps34 (PIK3C3) and ATG14 proteins in ETV6-RUNX1-bearing cell lines compared to other pre-B ALL or T-ALL cell lines as assessed by proteomics. (**d**) Expression levels of *pik3c3* and *atg14* mRNA in St. Jude cohort and EGA (European Genome-Phenome Archive) combined large dataset consisting of 419 pediatric ALL patients (ETV6-RUNX1 t(12;21), n = 18, other pre-B-ALL, n = 401). (**e**) Representative Western blotting (n = 3) to assess basal autophagic flux in ETV6-RUNX1 (REH and COG-LL-355h) compared to other cell lines (RCH-ACV, MHH-CALL2 and 697) as monitored by LC3B-II levels in the presence of bafilomycin A1, BafA1. GAPDH is used as loading control. The membrane was cut after transfer according to the corresponding protein sizes. Ratio of LC3B-II to GAPDH is quantified using Image J and presented under the figure. * *p* < 0.05; ** *p* < 0.01; *** *p* < 0.001, **** *p* < 0.0001 using either a two tailed t-test with Welch’s correction for two groups (**d**) and a 2-way ANOVA with Dunnett’s (one-to-many) multiple comparison test (**c**).

Using our recently published and publicly available data^15^, we assessed the activity of autophagy and lysosomal pathways in a panel of 41 Pre-B-ALL cell lines, based on transcriptomic and proteomic profiles. Using Principal component analysis (PCA) of RNAseq data, the cell lines were clustered according to their cytogenic background (Fig. 1a). Gene enrichment analysis (GSEA) showed that cell lines with ETV6-RUNX1 t(12:21) had activated autophagy pathway as compared to all other cell lines (Fig. 1b). Furthermore, analysis using DESeq showed that a large number of autophagy-related and lysosomal degradation-related genes were significantly more expressed in cell lines with ETV6-RUNX1 t(12;21) translocation than in other pre-B-ALL cell lines (Supplementary Fig. 1a, b). At the protein level, Vps34 and ATG14, both crucial for autophagy initiation, were expressed at significantly higher levels in cell lines bearing t(12;21) (Fig. 1c). Further, in a cohort of 419 pediatric ALL patients *PIK3C3* and *ATG14* mRNA were expressed at higher levels in the ETV6-RUNX1 t(12;21) subgroup (Fig. 1d). Using the online platform tool at https://www.proteomics.se/deepmeltome/.^16^ for analysis of proteoforms, we detected two main proteoforms of Vps34 at 41°C in most cell lines (Supplementary Fig. 1c, d). One of the Vps34 proteoforms (membership 1 in red color) was clearly more abundant in the ETV6-RUNX1 t(12;21) cell lines REH and COG-LL-355h as compared to other cell lines (Supplementary Fig. 1c), which was also associated with unphosphorylated peptides (covering the 163 and 165 active residues, Supplementary Fig. 1d for a linear and a 3D structure), suggesting a kinase activity characteristic for the autophagy-related Complex 1^17^. Finally, autophagic flux was investigated using Bafilomycin A1 (BafA1) in a smaller panel of pre-B-ALL cell lines with Western blotting for LC3B-II^18,19^. LC3B-II levels were higher in t(12;21)-bearing REH and COG-LL-355h cell lines as compared to two other cell lines (apart from RCH-ACV that has exceptionally high basal levels of LC3B-I protein (Fig. 1e. For the original images, see Supplementary Fig. 6a). Together, these data show a predominant upregulation of Vps34 and ATG14 and of the autophagy- and lysosomal degradation- related genes and pathways in leukemic cells with ETV6-RUNX1 t(12;21) translocation.

### Leukemic cells with t(12;21) translocation are sensitive to autophagy/lysosomal inhibition

**Figure 2:**
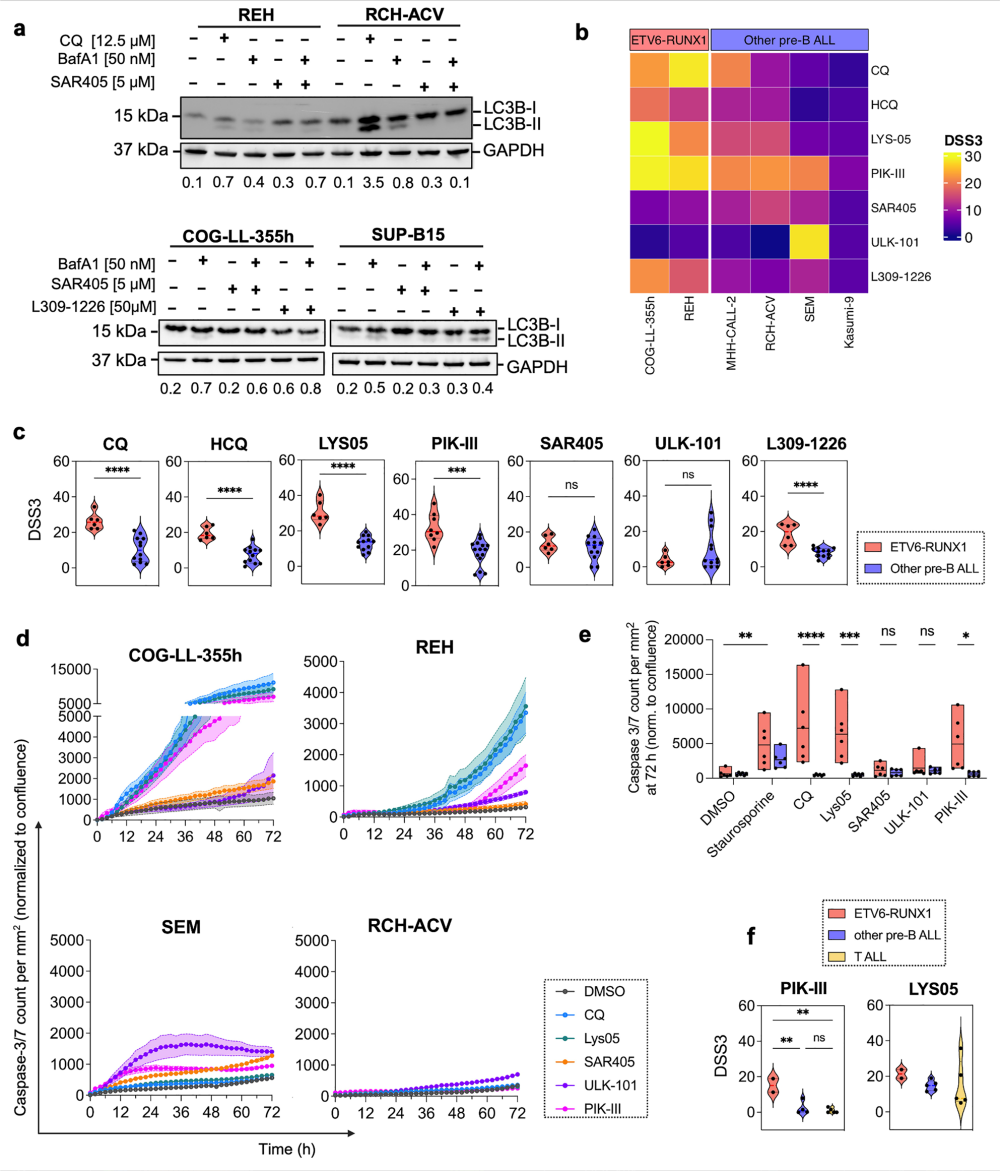
ETV6-RUNX1 ALL cells are particularly sensitive to autophagy inhibitors. (**a**, upper panel—one membrane) REH and RCH-ACV cells were treated with either CQ or SAR405 for 24 h in the presence or absence of BafA1 and LC3B-II levels were monitored by Western blotting. (**a**, lower panel) COG-LL-355 and SUP-B15 cells were treated with L309-1226 or SAR405 for 24 h in the presence or absence of BafA1 and LC3B-II levels were monitored by Western blotting on two separate membranes for each cell line. Representative image of three independent experiments is shown. GAPDH is used as a loading control. Each membrane that was cut after transfer according to the corresponding protein sizes. Ratio of LC3B-II to GAPDH is quantified using Image J and presented under the figure. (**b**, **c**) Indicated cell lines were treated with indicated autophagy and lysosomal inhibitors for 72 h and viability was assessed using CellTiter-Glo. DSS3 was calculated using DSRT online tool; higher DSS3 values indicate higher sensitivity. Data is presented as a heatmap (**b**) or violin plots (**c**) of individual experiments comparing the two ETV6/RUNX1 to four other pre-B-ALL cell lines (n ≥ 3). (**d**, **e**) IncuCyte live-cell imaging of cells treated with the indicated autophagy inhibitors for 72 h (n = 3). (**d**) caspase 3/7 activity is normalized to cell confluence and presented over time. Note the different Y-axis scale. Treatment with 0.1 µM Staurosporine was used as a positive control for apoptosis induction. (**e**) Quantification of mean relative caspase 3/7 levels at the 72 h time point ± SEM (n = 3, each performed in triplicate). (**f**) Primary ALL cells were cultured ex vivo and treated with indicated autophagy/lysosomal inhibitors in either 96 or 384-well plates for 48 h (n = 1 for 11 individual samples, each performed in triplicate). Cell viability was measured using CellTiter-Glo and drug sensitivity scores were generated using the DSRT online tool; higher DSS3 values indicate higher sensitivity. * *p* < 0.05; ** *p* < 0.01; *** *p* < 0.001, **** *p* < 0.0001 using either a two tailed t-test with Welch’s correction for two groups (**c**) or 2-way ANOVA with Šidák (**d**) or Tukey (**f**) multiple comparison tests.

Based on the data above, we asked whether increased levels of Vps34 and ATG14 as well as activity of the autophagy and lysosomal pathways in cells harboring the ETV6-RUNX1 t(12;21), can lead to a dependence on these proteins’ and pathways’ activity for cell survival. An initial automated high throuput drug testing assay using lysosomal inhibitors HCQ and Lys-01, and a Vps34i SAR405 at a wide range of concentrations showed a higher sensitivity of ETV6-RUNX1 cells for HCQ as compared to other pre-B ALL cell lines (Supplementary Table 2 and Supplementary Fig. 2a). Western blotting for LC3B-II verified that the inhibitors indeed inhibited the lysosomal or autophagy pathways: CQ led to the accumulation of LC3B-II, as an inhibitor of autophagosome-lysosome fusion; Vps34i SAR405 inhibited autophagic flux measured in BafA1-treated cells and manifested by a decrease in LC3B-II (Fig. 2a, most prominently in RCH-ACV). ATG14i L309-1229 should selectively block Vps34-complex I activity, and therefore autophagic flux; these effects were, however, less pronounced (Fig. 2a, lower panel).

We next fine-tuned the range of concentrations for each compound and cell line to avoid non-specific toxicities, and performed drug sensitivity assay in 96-well-plate format. We used water soluble dimeric CQ, Lys-05^20^ and also added a more potent Vps34i, PIK-III^7^, an ULK1/2 kinase inhibitor, ULK101, acting upstream of Vps34, and the above mentioned ATG14i, L309-1229^8^. The calculated drug sensitivity scores (DSS^21^) showed that ETV6/RUNX1 t(12;21) cell lines were more sensitive to CQ, HCQ and Lys-05, and to PIK-III and ATG14i than other pre-B-ALL cell lines (Table 1 and Fig. 2b, c). This was not the case, however, for SAR405 or ULK1/2i (Fig. 2b, c).

**Table 1:** Mean Drug Sensitivity Scores, DSS3, of pre-B-ALL cell lines to a panel of autophagy and lysosomal inhibitors (n = 3).

DRUG/Compound	COG-LL-355h	REH	MHH-CALL-2	RCH-ACV	SEM	KASUMI-9
Chloroquine	22.5	28.3	20.6	9.5	5.3	2.1
Hydroxychloroquine	19	13.4	11.1	10.1	1.7	4
LYS-05	29.5	21.1	15	15.4	6.4	5.3
PIK-III	29	27.6	21	22	20.7	7.6
SAR405	7	8.9	10.7	14.5	11	4.3
ULK-101	1.4	4.3	2.8	0	28.1	4.4
L309-1226	21.6	16.5	9.4	7.2	11.1	4.8

Higher DSS values indicate higher sensitivity.

We then assessed apoptotic cell death using caspase 3/7 reagent and IncuCyte live-cell imaging over time (Fig. 2d, e and Supplementary Fig. 2b, c for cell confluence). Lysosomal inhibitors CQ and Lys-05, and Vps34i PIK-III strongly induced cell death in the two ETV6/RUNX1 t(12;21) cell lines but not in the two other cell lines (Fig. 2d, compare upper to lower graphs, note difference in values on Y-axis; Fig. 2e for quantification). Finally, ex vivo treatment of primary patient-derived leukemic cells of different cytogenetic background with lysosomal and Vps34 inhibitors showed that cells from the two ETV6/RUNX1 t(12;21) bearing samples were significantly more sensitive to PIK-III as compared to cells from other pre-B-ALL genetic sub-groups or T-ALL (Fig. 2f and Supplementary Tables 3 and 4 for patient information and DSS scores, respectively). Together these experiments showed that ETV6/RUNX1 t(12;21)—bearing leukemic cells were more sensitive, although with some variation, to the lysosomal or Vps34 inhibition, as compared to cells from other genetic sub-groups. In overall, the Vps34i PIK-III was superior to all other drugs in preferential killing of t(12;21)—bearing leukemic cells.

### Inhibitors of autophagy and Dexamethasone synergize in induction of cell death

Apart of being activated in cancer cells, autophagy is also induced by the cytotoxic drugs. Previously, we have shown that Dexamethasone (Dexa), a cornerstone drug used in ALL treatment, induced profound autophagy in ALL cells^13,14^. As autophagy is often a cytoprotective mechanism in cells, we asked whether combination of Dexa with autophagy inhibition could potentiate cell death of ALL cells.

**Figure 3:**
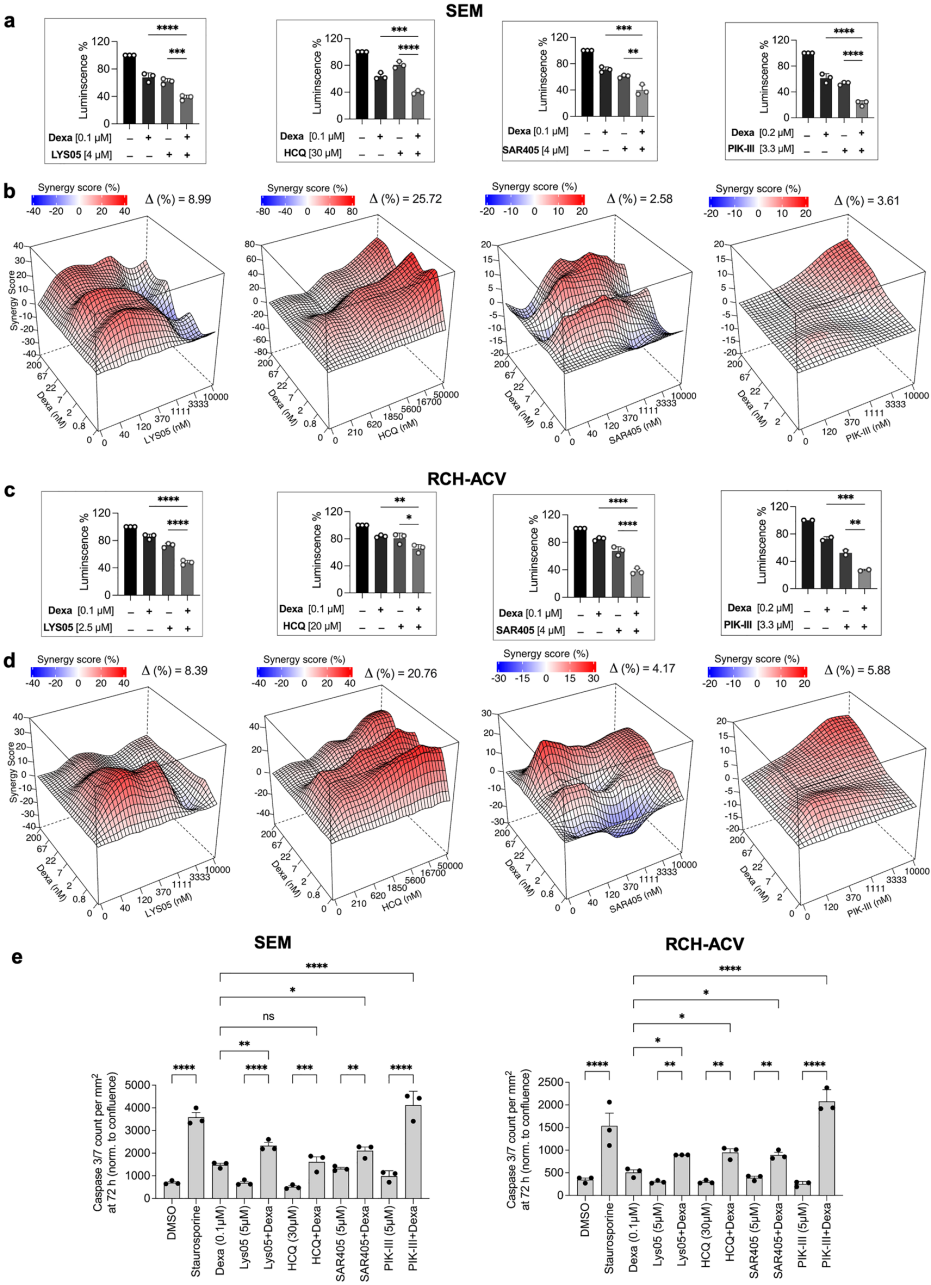
Combination of autophagy inhibitors with dexamethasone show synergistic effects in pre-B-ALL cells. (**a** and **c**) SEM (a) or RCH-ACV (**c**) cells were seeded on 96 well plates for 24 h and treated with indicated drugs and drug combinations for 72 h; viability was measured using CellTiter-Glo (n = 3, each performed in triplicate). The mean ± SD is shown. (**b** and **d**) SEM (b) or RCH-ACV (d) cells were seeded in automated manner into 384 well plates containing indicated drug dilutions and their combinations, besides Dexa + PIK-III, which was done manually in 96-well plates as in a,c), and incubated for 72 h, and cell viability was measured using CellTiter-Glo. Data were analyzed using SynergyFinder. Representative two-drug interaction surface plots for the indicated concentration ranges from two independent experiments are shown. (**e**) IncuCyte live-cell imaging of SEM or RCH-ACV cells treated with the indicated drugs and drug combinations for 72 h (n = 3, each performed in triplicate). Green fluorescence corresponding to caspase 3/7 activity at the 72 h time point was normalized to cell confluence and presented ± SEM. Treatment with 0.1 µM Staurosporine was used as a positive control for apoptosis induction. * *p* < 0.05; ** *p* < 0.01; *** *p* < 0.001, **** *p* < 0.0001 using one-way ANOVA with Holm-Šidák multiple comparison test.

**Figure 4:**
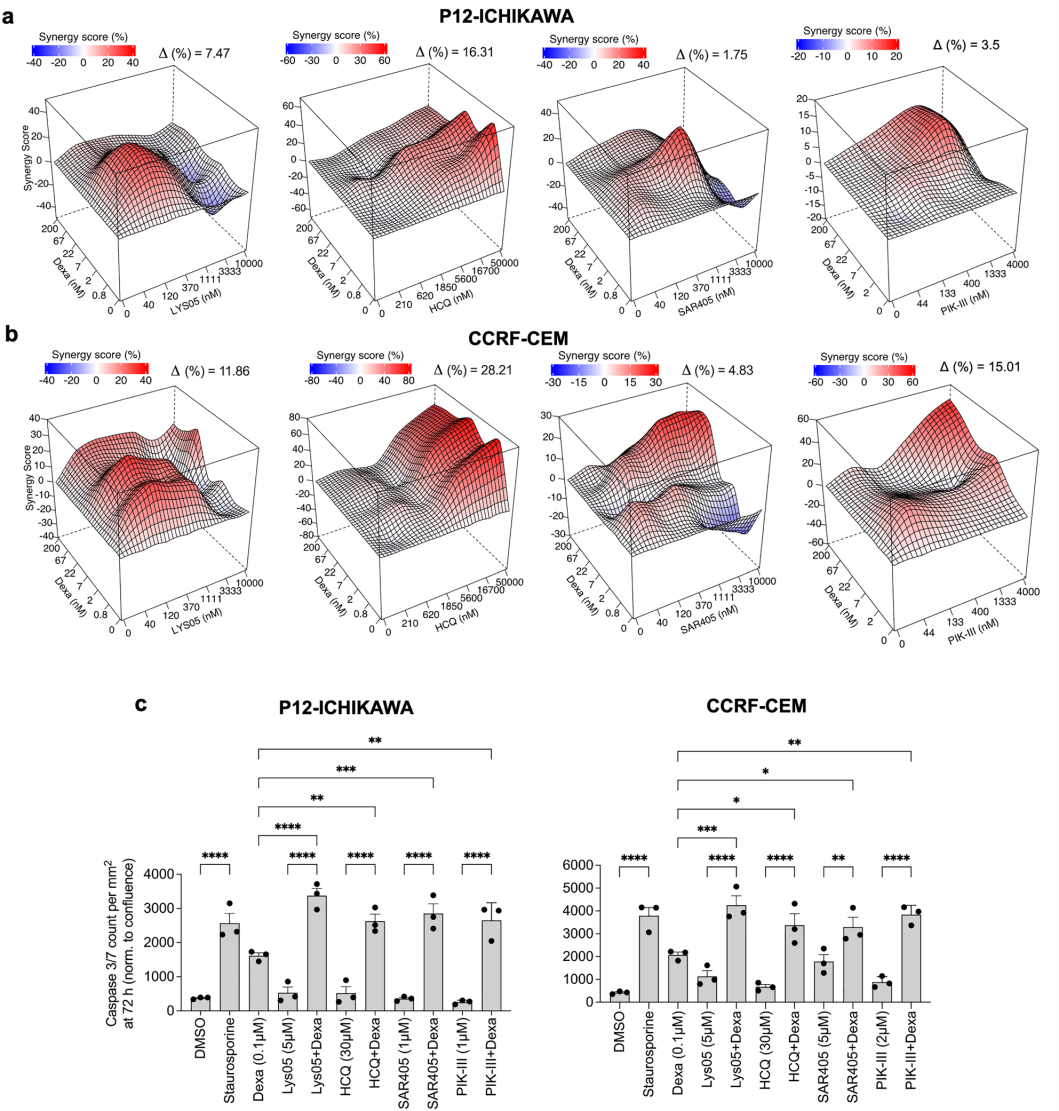
Combination of dexamethasone and autophagy inhibitors show synergistic effects in T-ALL cells. (**a**, **b**) P12-Ichikawa (**a**) and CCRF-CEM (**b**) cells were seeded in automatic manner into 384 well plates containing indicated dilutions of drugs and their combinations (besides Dexa + PIK-III combination, which was done manually in 96-well plates as in Fig. 3a, c), incubated for 72 h and cell viability was measured using CellTiter-Glo. Data was analyzed using SynergyFinder. A representative two-drug interaction surface plot for each cell line is shown (n = 2). (**c**) IncuCyte live-cell imaging of P12-Ichikawa and CCRF-CEM cells treated with the indicated drugs and drug combinations for 72 h (n = 3, each performed in triplicate). Green fluorescence corresponding to caspase 3/7 activity at 72 h time point was normalized to cell confluence and presented ± SEM. Treatment with 0.1 µM Staurosporine was used as a positive control for apoptosis induction. * *p* < 0.05; ** *p* < 0.01; *** *p* < 0.001, **** *p* < 0.0001 using 1-way ANOVA with Šidák multiple comparison test.

Both the Western blotting and transcriptome analysis of previously published data^15^ revealed an absence of glucocorticoid receptor, GCR (NR3C1), in REH cells, low levels in Kasumi-9 cells, with moderate to high levels of expression in other cell lines (Supplementary Fig. 3a, b, respectively). GCR moved to the nucleus upon treatment with Dexa in cell lines with both the high or low levels of expression demonstrating a functional GCR (data not shown). There was also a correlation between GCR (NR3C1) protein expression and sensitivity to Dexa in our previously published data^15^ (Pearson correlation coefficient = 0.75, Supplementary Fig. 3c). Using this data, we have chosen five pre-B-ALL (Fig. 3) and five T-ALL (Fig. 4) cell lines, based on their moderate levels of GCR expression and intermediate sensitivity to Dexa, and performed combination treatments using Dexa and either lysosomal (HCQ, Lys-05) or autophagy inhibitors (SAR405, PIK-III) (for ZIP scores, see Table 2 and Supplementary Fig. 3d). Combination of Dexa with each of the inhibitors significantly decreased SEM and RCH-ACV cell viability as compared to single treatments (Fig. 3a and c, respectively). High throughput combination treatments for the five pre-B-ALL and five T-ALL cell lines showed universally high synergy between Dexa and lysosomal inhibitors Lys-05 or HCQ in all cell lines (Table 2, Fig. 3b, d and Fig. 4 a, b and Supplementary Fig. 4). Notably, synergy between these drug pairs is achieved even at lower drug concentrations. Synergy between Dexa and the Vps34is SAR405 or PIK-III was weaker as a rule with superior effects of PIK-III over SAR405 (Table 2, Fig. 3b, d and Fig. 4a, b, Supplementary Fig. 3d).

**Table 2:** Mean ΔZIP synergy scores for drug combinations of pre-B-ALL and T-ALL cell lines (n = 3).

Drug combination	Pre-B-ALL					T-ALL				
	SEM	RCH-ACV	LC4-1	NALM-6	697	P12-ISCHIKAWA	KARPAS-45	CCRF-CEM	CCRF-HSB2	ALL-SIL
Lys05-Dexamethasone	8.96	8.45	12.4	8.99	8.05	7.51	8.75	11.86	9.53	9.34
SAR405-Dexamethasone	2.7	4.13	8.39	8.0	2.25	1.77	0.75	4.9	1.68	0.64
HCQ-Dexamethasone	25.47	20.77	52.13	34.36	16.65	16.23	8.23	28.33	4.9	15.26
PIKIII-Dexamethasone	3.61	5.88	N/A	N/A	N/A	3.5	N/A	1.6	N/A	N/A

We then studied apoptosis in pre-B-ALL cell lines SEM and RCH-ACV, and T-ALL cell lines P12-ICHIKAWA and CCRF-CEM treated with all drug combinations over time monitored by Caspase 3/7 activation and live-cell imaging analysis (Figs. 3e and 4c, respectively, and Supplementary Fig. 5 for cell confluence and an image example). Data showed significantly higher number of apoptotic cells in the combination as compared to single treatments in either cell line and for each of the drugs. The data suggests that both lysosomal and autophagy inhibition potentiates apoptotic cell death induced by Dexa in ALL cells.

**Figure 5:**
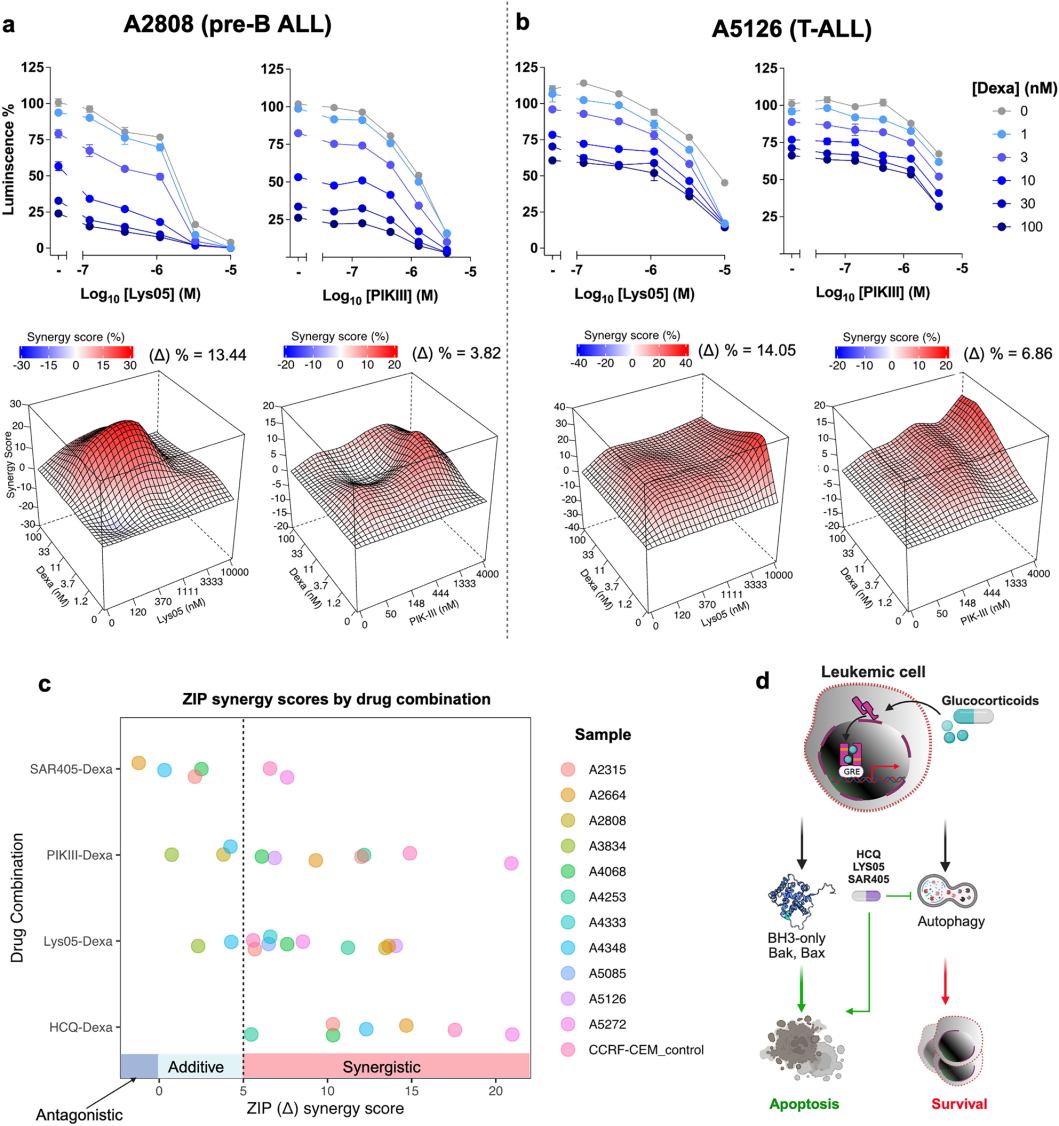
Combination of dexamethasone and autophagy inhibitors show synergistic effects in ALL primary cells ex-vivo. (**a**, **b**) Primary ALL cells were seeded either in 384 (in duplicate) or in 96-well (in triplicate) plates (n = 11, 6 Pre-B- and 5 T-ALL) and treated with either Dexa alone or in combination with Lys-05 or PIK-III for 48 h and viability was measured using CellTiter-Glo. A representative of a pre-B- (**a**) or a T-ALL (**b**) sample; data shown as inhibition of viability curves (upper) or as two-drug interaction surface with respective ZIP synergy scores (lower); (**c**) Summary of data of ZIP scores (X-axis) of the indicated drug combinations (Y-axis) for the 11 primary samples (Table 3 for ZIP scores and Supplementary Table 3 for patient information). (**d**) Schematic illustration of Dexa-induced leukemia cell death and potentiation through inhibition of autophagy or lysosomal degradation.

Finally, we tested translatability of our findings in primary ALL blasts isolated from bone marrow (BM) or peripheral blood (PB) of patients with ALL at diagnosis or relapse (6 pre-B-ALL and 5 T-ALL, see Supplementary Table 3). There were synergistic or additive effects between Dexa and HCQ, Lys-05, SAR405 or PIK-III drug-combinations on a majority of samples without any significant difference within the genetic sub-groups (Table 3 and Fig. 5a, b, c). HCQ, Lys-05 and PIK-III showed the best scores (Fig. 5c).

**Table 3:** Mean ΔZIP synergy scores for the drug combinations of ALL patient samples (n = 3).

Drug combination	Pre-B-ALL						T-ALL				
	A5085	A2808	A4068	A4348	A5272	A4333	A3834	A4253	A2315	A2664	A5126
Lys05-Dexamethasone	6.5	13.44	7.6	4.28	8.54	8.45	2.31	11.21	5.68	13.63	14.05
SAR405-Dexamethasone	N/A	N/A	2.51	0.31	7.6	N/A	N/A	N/A	2.12	− 1.22	N/A
PIKIII-Dexamethasone	N/A	3.82	6.1	4.23	20.92	N/A	0.74	12.17	12.02	9.3	6.68
HCQ-Dexamethasone	N/A	N/A	10.33	12.3	20.98	N/A	N/A	5.47	10.32	14.68	N/A

Taken together, our results show that inhibition of lysosomal/autophagy pathways enhanced the cytotoxic effects of GCs in ALL blasts, and inhibitors of these pathways can be used to sensitize to GCs and even overcome GC resistance in ALL (a schematic hypothesis in Fig. 5d).

## Discussion

In this pre-clinical study we have shown that inhibition of autophagy by either lysosomal or more selective autophagy inhibitors can be beneficial for the treatment of pediatric acute leukemia. We have studied this using two different approaches: (1) drugs that can be used as precision therapeutics in a particular genetic sub-group of ALL and (2) drug candidates for a combination therapy with GCs.

Pediatric ALL divided into immunological sub-types of pre-B-ALL and T-ALL is a genetically heterogenous disease with more than 10 genetic subgoups^9^ and, therefore, we asked if any of these subgroups can be selectively dependent on the autophagy or lysosomal pathways. We have analyzed RNAseq and proteomics data from our recent studies on large collection of cell lines^15,16^ and used existing drugs, such as lysosomal inhibitor HCQ, more potent derivative Lys-05^20^ or novel selective compounds targeting autophagy^4,5,7^. We found high levels of Vps34 and autophagy in leukemic cells of ETV6-RUNX1 t(12;21) genetic subgroup and a higher sensitivity of these cells to the lysosomal inhibitors, as compared to other genetic subgroups, being in line with one previous publication that showed a dependence of ETV6-RUNX1 bearing cells on Vps34^11^. Also, ATG14 protein^22^ was highly expressed, and one of the Vps34 proteoforms was enriched in cells of this genetic subgroup. We further showed that targeting just Vps34 or ATG14, both required for autophagy initiation, can be beneficial in this particular genetic subgroup. There were variations in the efficiency of the inhibitors. One of the Vps34is, PIK-III, was more effective suggesting that it might be a matter of potency or a more selective activity. While higher levels of Vps34 in ETV6-RUNX1 cell lines require higher drug concentrations for a complete inhibition of Vps34, a more potent Vps34i, such as PIK-III^7^, has stronger effects. Notably, we could show a superior effect of PIK-III also in t(12;21) primary leukemic cells collected at diagnosis as compared to cells of other genetic sub-groups or T-ALL. With regard to the ATG14i, L309-1226, that blocks protein–protein interaction between Beclin1 and ATG14L^8^, no inhibition of autophagic flux was observed in RCH-ACV or Sup-B15 cells (as in case of Vps34i SAR405 by measuring LC3B-II formation in the presence of BafA1). ATG14 is initially localized to the phagophore or isolation membrane in a PI3-kinase Complex I together with co-binding proteins^2,22^. However, in addition, ATG14 oligomers can to be recruited to facilitate autophagosome fusion with lysosomes at the late stages of autophagy^23^. Thus, L309-1226 can also block autophagy at later stages resulting in accumulation of LC3B-II (rather than inhibiting its formation), which was exactly what we have observed. Taken together, our data suggest that lysosomal and autophagy inhibition could be an efficient way to selectively target ETV6-RUNX1 bearing leukemic cells. Since over-treatment is one of the problems of the current treatment protocols, and ETV6-RUNX1 genetic subgroup is usually associated with a favorable prognosis, the treatments based on biomarkers are warranted, and our work may represent such a precision medicine opportunity in ALL.

Many studies demonstrated that various cytotoxic drugs used in anti-cancer treatment can induce cytoprotective autophagy^1,4^, leading to clinical trials combining these anti-cancer agents with lysosomal drugs. We previously found that Dexa, uniformly used in the treatment of ALL, induces profound autophagy, which is required for the onset of apoptosis^13^. The mechanism of this was unknown, but we speculate that it involves signaling pathways (such as production of a pro-apoptotic cleaved form of ATG-5 or down-regulation of BCL2 family members) rather than autophagic cell death. This is supported by the cell death mechanism by caspase-dependent apoptosis^24^, and by the observation that accelerating autophagy by either mTOR inhibition or amino acid starvation did not result in any further increased cell death^13^. There are publications on both the pro-survival and pro-death effects of autophagy in leukemias^25^, and the reasons are not well understood. Therefore, we decided in this study to expand to more cell lines, including T-ALL, and primary ALL cells, and to block autophagy at both the initiation stage with more selective autophagy inhibitors and at the later stage of lysosomal degradation. This approach was also motivated by our recent findings describing upregulation of the lysosomal pathway by Dexa^14^. Our data clearly showed a strong synergy between Dexa and lysosomal drugs in inducing loss of viability and induction of apoptosis in both pre-B- and T-ALL, independently of their genetic background. A less pronounced but clear either synergistic or significant additive cytotoxic effects of Vps34is were observed. Thus, we can conclude that while some initial signaling leading to autophagy induction might be necessary for cell death, the profound autophagy induced by Dexa primarily has cytoprotective effects. Similar data using CQ were obtained in a most recent study that also showed induction of autophagy and mitophagy in Dexa-treated T-ALL cells^26^. Another treatment modality in pediatric ALL is L-Asparaginase (Asp), which also induced autophagy, and the combination of Asp and CQ was synergistic in ALL cell lines^11^. These data together suggest that autophagy inhibition can be beneficial in combination with existing therapeutic modalities in pediatric ALL treatment.

Another important question to discuss is whether elevated autophagy may underlie GC resistance, and whether treatment with lysosomal or selective autophagy inhibitors could alleviate such resistance to GCs in ALL. GCs are especially important since GC-resistance is a prognostic factor in childhood ALL^12^, representing important point of failure impacting patients during treatment. We and others have found a correlation between levels of GC receptor (GCR or NR3C1) expression and sensitivity to Dexa^15^. Thus, low levels of GCR may represent a biomarker for combination treatment with lysosomal inhibitors. In this study, we have chosen cell lines with intermediate levels of GCR and intermediate sensitivity to Dexa and showed that it is possible to potentiate Dexa-induced cell death by autophagy inhibition in such cells. On the other hand, several other reasons for GC resistance have being described, such as impaired apoptosis^27^, metabolic reprograming towards elevated carbohydrate metabolism^28^ or increased fatty acid oxidation^26^. In a recent study, increased autophagy/mitophagy was linked to acquired resistance to Dexa, since autophagy was cytoprotective in the resistant to Dexa cells^26^. We, however, found in the present study that autophagy inhibition can further potentiate cytotoxicity even in cells initially sensitive to GCs, such as in patient-derived primary ALL cells.

The relationship between metabolic reprograming in response to Dexa and autophagy induction remains to be studied. We and others have found that Dexa treatment leads to an inhibition of glucose uptake and metabolism and a switch from glycolysis and glutaminolysis to a fatty acid oxidation^26,29^. However, while neither low glucose not 2-DG induced or synergized with autophagy induction^29^, low glutamine levels in media accelerated Dexa-induced autophagy, suggesting that availability of glutamine and also production of ammonia may underlie induction of autophagy by Dexa^14^. Certainly, recently described Dexa targeting to mitochondria, mitochondria damage and ROS production in response to Dexa is another plausible mechanism^26^. Future studies using a reliable and fast method to measure autophagy in primary leukemic cells at diagnosis and correlate autophagy to Dexa sensitivity ex vivo can help answering the question of the role of autophagy in GC resistance in leukemic cells.

In conclusion, our present study together with previous research strongly suggests the use of autophagy inhibition for combination therapy to overcome or even to prevent therapy resistance to such important drugs as GCs in ALL. Keeping in mind that high doses of GCs for the treatment of children with ALL has severe side effects that can be devastating and long-lasting^9^, combining GCs with lysosomal or autophagy inhibitors might make it possible to lower the doses and thus alleviate these side effects.

### Significance

In the present study, we used global proteomics and transcriptomics-based approaches and drug testing, to identify autophagy/lysosomal pathways as valid therapeutic targets in a subgroup of pediatric ALL. Further, combining autophagy inhibition with glucocorticoids, cornerstone drugs in the ALL treatment, resulted in synergistic cytotoxic effects in both the cell lines and primary leukemic cells. Thus, our data provide a basis for targeting autophagy/lysosomal pathways in ALL treatment and thus take a step towards personalized treatment of pediatric patients with ALL.

## Methods

### Cell culture and treatments

#### Cell culture

Human leukemia-derived cell-lines with indicated chromosomal rearrangements or genetic abberrations used in this study are summarized in Supplementary Table 1 and our recent study^15^. RPMI 1640 (#R2405, Sigma Aldrich, Solna, Sweden) or Iscove’s Modified Dulbecco’s Medium (Cat.# I2911, Sigma Aldrich) medium were supplemented with 10% or 20% of heat-inactivated fetal bovine serum (FBS) (Cat.# F9665 Gibco), 25 mM HEPES (Cat.# 15630080 Gibco) and 1% penicillin–streptomycin (Cat.# 10378016 Gibco) and 1% L-Glutamine (Cat.# A2916801 Gibco). 1% Insulin–transferrin–sodium selenite (ITS) (Cat.# I3146 Sigma Aldrich) was used for COG-LL-355h. All cell lines were maintained in a humidified incubator with 5% CO_2_ at 37 °C.

The following compounds were used for drug testing and combination treatments: Dexamethasone (Cat.# D4902 Sigma Aldrich), Chloroquine (Cat.# S4157 Selleckchem), Hydroxychloroquine (Cat.# H0915 Sigma Aldrich), LYS-01 (kindly provided by Dr. Amaravadi), LYS-05 (Cat.# S8369 Selleckchem), SAR405 (Cat.# S7682 Selleckchem), PIK-III (Cat.# S7683 Selleckchem), ULK-101 (Cat.# S8793 Selleckchem), L309-1226 (ChemDiv) and Bafilomycin A1 from *Streptomyces griseus* (Cat.# B1793 Sigma Aldrich). The compounds were purchased in solid form and reconstituted in 100% dimethyl sulfoxide (DMSO) (Cat.# D8418 Sigma Aldrich), except for Chloroquine and Hydrochlroquine which were dissolved in water. For long-term storage, the compound stocks were aliquoted and kept at − 20 °C.

### Publicly available gene expression and thermal proteome profiling data

Bulk RNA-seq based gene expression data of 41 pre-B ALL cell lines published by us^15^ was retrieved from NCBI’s Gene Expression Omnibus accessible through GEO Series accession number GSE168386. Proteomics-based gene expression data from that study were dowloaded from the ProteomeXchange Consortium via the PRIDE partner repository using the dataset identifier PXD023662. As described in our previous publication, a dataset was assembled including transcriptomic data for a total of 417 leukemia samples from pediatric patients obtained from The European Genome-phenome Archive (EGA) (Dataset ID: EGAD00001002704 and EGAD00001002692) after Data Access Agreement (DAC) approval from St. Jude Children’s Research Hospital—Washington University Pediatric Cancer Genome Project Steering Committee, as well as two additional RNA-seq samples of MEF2D-HNRNPUL1 subtype obtained from the Shanghai Institute of Hematology (SIH) processed together with the St. Jude cohort^15^. Differential gene expression analysis was performed on the RNA-seq data using the R Bioconductor package edgeR^30^ implemented in a shiny app or using the DESeq2 R package^31^. Gene enrichment analysis was also performed in R with Over-Representation Analysis (ORA) using log fold changes (logFC) of core:upregulated genes between the ETV6-RUNX1 subtype and other pre-B ALL cells. Data were filtered to include only genes with logFC > 0.3, *P* value < 0.05, log counts per million (logCPM) > 1, and the false discovery rate was set as fdrThr = 0.05. Comprehensive pathway enrichment analysis was performed using the CRAN-based WebGestalt R package^32^ using either the Gene Ontology Biological Process (GO:PB)^33^ or Kyoto Encyclopedia of Genes and Genomes (KEGG) pathway enrichment databases^34^.

The relative abundances and melting profiles of Vps34 protein complexes were assessed using Thermal Proteome Profiling (TPP) as described in our recent study^16^. TPP data for VPS34 was retrieved, also available in raw form on PRIDE with the dataset identifier PXD031162 and browsable at https://www.proteomics.se/deepmeltome/. In brief, peptide mappings were obtained with Leiden-assigned clustering at resolution 1. Domain descriptions, including modification sites, were obtained from Uniprot under the accession Q8NEB9. Peptide mappings and coordinates were assigned for all detected peptides from the FASTA sequence associated with this accession, and the structural visualization plot was downloaded from https://alphafold.ebi.ac.uk/ as generated using the AlphaFold Monomer v2.0 pipeline.

### Cell viability and drug combination assay

Cells were seeded at 2 × 10^5^ cells/mL in 96-well flat-bottom white plates (Cat.# 136101, Thermo Fischer) for all cell lines except for COG-LL-355h cell line, which was seeded at 4 × 10^5^ cells/mL. Cells were incubated at 37 °C and 5% CO_2_ in a humidified incubator for 24 h before treatments. The DMSO levels were normalized across the plate at a maximum of 0.1%. Drugs were serially diluted with concentrations ranging from 0.3 to 10 µM for Vps34 inhibitors and 1.5 to 50 µM for lysosomal inhibitors in a 96-well plate format. Cells were incubated with drugs/compounds for 72–96 h, before measuring cell viability using CellTiter-Glo 2.0 ATP reagent (Promega) on Varioscan LUX microplate reader. Relative cell viabilities were subsequently determined by normalizing to cell-only (100% viability) and medium-only (0% viability) wells and further curve-fitted using a nonlinear regression model (variable slope, four-parameter, GraphPad Prism) when applicable to determine IC50 values. To establish drug sensitivity score (DSS^21^) values, a standardized Drug Sensitivity and Resistance Testing (DSRT) tool^35^ was used.

For high throughput assays, compounds were pre-spotted onto 384-well plates (Greiner) using an Echo 550 acoustic dispensing system (Beckman Coulter). When applicable, the DMSO levels were normalized across the plate at a maximum level of 0.1%. Cells were then seeded into the compound-containing plates using a Multidrop Combi Reagent Dispenser (Thermo Fisher Scientific), at 4 × 10^5^ cells/mL in 50 μL medium. Cells were then incubated at 37 °C and 5% CO_2_ in a humidified incubator for 72 h, before measuring cell viability using CellTiter-Glo on either Varioscan LUX (Thermo Fisher Scientific) microplate reader or EnSight (PerkinElmer) microplate reader.

### IncuCyte live-cell imaging

Clear flat-bottom 96-well plates (Cat.# 3599, Corning) were coated with 0.01% Poly-L-ornithine solution (Cat.# P4957, Thermo Fischer Scientific, Stockholm, Sweden) for 24 h at 37 °C, washed with PBS and air dried for 30 min before cells were seeded at 2 × 10^5^ cells/mL (4 × 10^5^ cells/mL for COG-LL-355h) in 100 µL of appropriate complete growth medium. 24 h later both the compounds for treatment and 1 μM of Cellevent Caspase-3/7 Green Dye (Cat.# C10723, Thermo Fischer Scientific) was added to cells. 0.1 µM of staurosporine was used as a positive control for apoptosis induction in every experiment. Phase-contrast and green fluorescence images (10X) were acquired using IncuCyte (Sartorius) every 2 h for 72 h, and cell confluence (%) was calculated using the IncuCyte S3 live-cell analysis system (Sartorius). Caspase 3/7 induction was calculated as per cent of green fluorescent object counts normalized to cell confluence.

### Western blot and antibodies

Cell pellets were lysed in freshly prepared modified RIPA buffer (50 mM Tris–HCl pH7.4, 150 nM NaCl, 1 mM EDTA, 1% NP-40, and 1% glycerol) containing protease inhibitor cocktail and phosphoSTOP (Roche). The protein concentration of the extracts was measured using a bicinchoninic acid (BCA) assay (Thermo Fischer Scientific). A total of 30 µg of the proteins were separated on 4–12% or 12% Bis–Tris gels (NuPAGE, Life Technologies), followed by transfer on a methanol-activated polyvinylidene fluoride (PVDF) membrane. Membranes were then cut according to protein sizes (for LC3B, GAPDH, GCR or β-actin), blocked with 5% skimmed milk and incubated with respective primary antibodies diluted in blocking agent overnight at 4 °C, followed by a 1-h incubation with secondary antibodies at room temperature. Proteins were detected using Western Lightening Plus-ECL (PerkinElmer) and visualized using iBright FL1000/ Invitrogen and iBright FL1000 Imaging system software (ThermoFisher Scientific). Ratios of LC3B-II to GAPDH or to β-actin were quantified using Image J and presented under each figure.

The following primary antibodies from Cell signalling Technology were used: anti-LC3B-II (Rabbit, D11, Cat.# 3868S); Anti-GCR (Rabbit mAb D6H2L XP, Cat.# 12041). Anti-β-Actin (Rabbit, Cat.# 4967). Anti-GAPDH was from Santa Cruz Biotechnology (Mouse, 0411, Cat.# sc-47724). Secondary antibodies were: Goat anti-rabbit IgG HRP-linked, Cell Signalling Technology Cat.# 7074, Goat anti-mouse Ig (H + L) HRP, Fischer Scientific, Cat# 626520.

### Primary ALL samples and cell culture

#### Isolation of mononuclear cells

Leukemic cells were obtained from bone marrow or peripheral blood from patients with primary ALL at diagnosis or relapse. Mononuclear cells were obtained by density gradient centrifugation using SepMate™ tubes (Cat.# 15450 STEMCELL Technologies), according to the manufacturer’s instructions. Isolated leukemic cells were cryopreserved in liquid nitrogen in FBS/10% DMSO.

#### Patient samples

The study included leukemic cells from patients diagnosed with precursor-B-ALL or T-ALL according to the World Health Organization classification^36^, for details see Supplementary Table 3. All patients and their parents were informed of the investigative nature of this study, and informed consent was obtained from each patient/parent in accordance with the ethical committee requirements; the experimental protocols for isolation of mononuclear cells from blood and bone marrow, for their long-term storage and usage in the methods described were approved by the following decisions: 01-069, amendment 2021-02718; 02-445 amendment 2018/38-32, Swedish Ethical Review Authority (Etikprövningsmyndigheten), Stockholm, Sweden. All experiments were performed according to Karolinska institutet’s guidelines and regulations; risk assessment for work with blood and other human materials were made in HUMRA according to institutets rules, and the experimental protocol was approved by the group leader.

#### Culturing protocol, drug treatment and cell viability

For the treatments, primary cells were washed, counted and seeded in Iscove’s modified MDM (Sigma Aldrich, I2911) containing 20% FBS, 1% of ITS Liquid Media Supplement (Sigma Aldrich) 1% L-Glutamin and 1% of penicillin–streptomycin in either 96-well plates or in 384-well plates, as described for cell lines, and 3 h later were treated with drugs for 48 h. Cell viability was assessed using CellTiter-Glo 2.0 (Promega).

### Data analysis

Graphs and statistical analyses were performed using GraphPad (Prism). Comparisons between groups were performed using two-tailed unpaired t-tests or one-way analysis of variance to compare three or more groups. *P* values of < 0.05 (*), < 0.01 (**), < 0.001 (***), < 0.0001 (****) were determined to be statistically significant. Sample size (n) refers to biological replicates. Synergy between two drug pairs was determined using the SynergyFinder package version 2.4 in R^21^ and values for Zero interaction potency (ZIP) were used. Based on ZIP synergy scoring model, a delta (Δ%) score below zero antagonistic, between 0 and 5 is additive and above 5 is synergistic.

## Data availablity

The datasets generated during the current study are available from the corresponding author on request. Data originating from outside of this study are appropriately referenced and publicly available from that source.

### Supplementary Information

Supplementary Information.
