# Supplementary material for: Targeting autophagy as a therapeutic strategy in pediatric acute lymphoblastic leukemia
Source: Sci Rep. 2024 Feb 18;14:4000. doi: 10.1038/s41598-024-54400-6 (PMC10874937; doi:10.1038/s41598-024-54400-6)
Supplement: Supplementary file 1 — Supplementary Information. [file 41598_2024_54400_MOESM1_ESM.docx]

**SUPPLEMENTARY MATERIAL**

**SUPPLEMENTARY FIGURES**


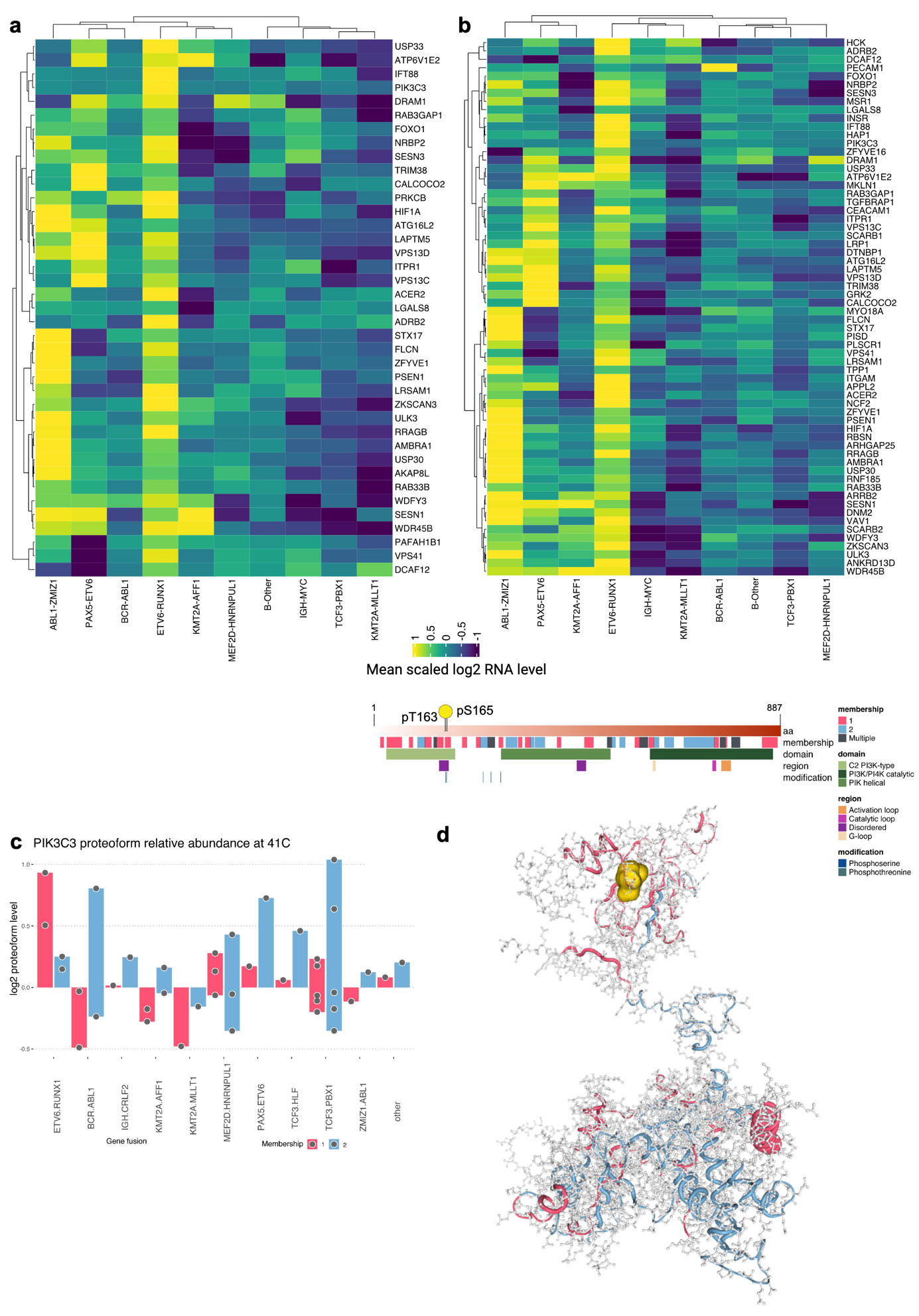


**Supplementary Figure 1: Activation of autophagy and lysosomal pathways in pre-B-ALL cell lines with ETV6-RUNX1 translocation.** (**a**) Differential expression of autophagy and **(b**) lysosomal genes in the indicated genetic subgroups of ALL; mean scaled log2 RNA levels (Clustering distances= Pearson, Clustering method = Ward.D2). (**c,d**) Analysis of Vps34 proteoforms. **(d)** Diagram of proteoform assignments, illustrating linear (top) and three-dimensional (bottom) models, with proteoform memberships labeled in red and blue, and their relative abundance as quantified in (**c)**. Data was retrieved and analyzed from the online tool at <https://www.proteomics.se/deepmeltome/>.The labeled domains and peptide coordinates represent data from the Q8NEB9 FASTA and features as annotated in Uniprot, and the structural model was computationally modeled using Alphafold. Yellow structure residues indicate the 163 and 165 phosphorylation sites. The grey backbone represents the full structure, and the colored ribbon segments represent the overlay of predicted 3D coordinates for proteoform-mapped peptides.

**
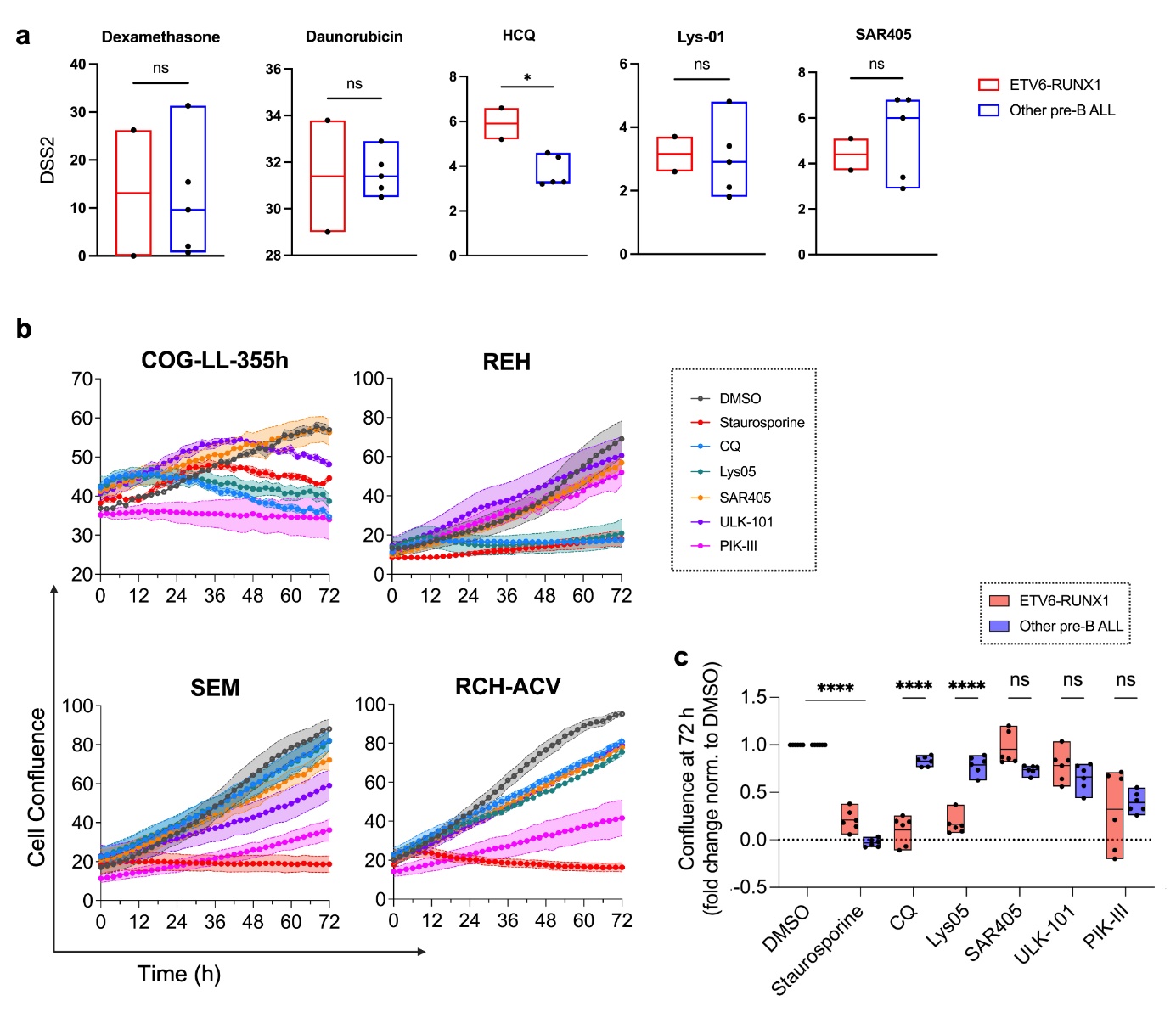
**

**Supplementary Figure 2.**

**ETV6-RUNX1 cell lines are more sensitive to lysosomal inhibitors as compared to other pre-B-ALL cell lines**. (**a**) Initial high throughput drug testing in a panel of pre-B-ALL cell lines. Plots compare ETV6-RUNX1 t(12;21) and other pre-B-ALL cell lines. DSS2 scores were calculated using the DSRT online tool (Supplementary Table 2). Higher DSS2 values indicate higher sensitivity. (**b**) Graphs presenting per cent of confluence of COG-LL-355 and REH (upper panel) and SEM and RCH-ACV (lower panal) after treatment with indicated inhibitors in kinetics using IncuCyte live-cell imaging. (**c**) Graphs presenting per cent of confluence for cell lines in (b) at 72 hr of treatment with indicated inhibitors.

**Supplementary Figure 3: Expression of glucocorticoid receptor and synergy between dexamethasone and autophagy inhibitors in ALL cell lines.** (**a**) GCR expression levels in a panel of pre-B-ALL cell lines as detected by Western blotting. The data are representative of two independent experiments with similar results. (**b**) mRNA expression levels of GCR (NR3C1) detected by RNA-Seq ^1^. (**c**) Sensitivity of a large panel of pre-B-ALL cell lines to dexamethasone plotted against GCR (NR3C1) mRNA expression levels showed a strong Pearson’s correlation (Pearson’s coefficient = 0.75, *p =* 5.5 e^-09^) ^1^ and analyzed using our on-line tool [FORALL (proteomics.se)](https://proteomics.se/forall/). Cell lines (highlighted in the figure) were selected for a synergy testing based on their moderate levels of GCR expression and an intermediate sensitivity to dexamethasone. **(d)** Summary of ZIP synergy scores (X-axis) of indicated drug combinations (Y-axis) on all the pre-B-ALL and T-ALL cell lines used in this study.


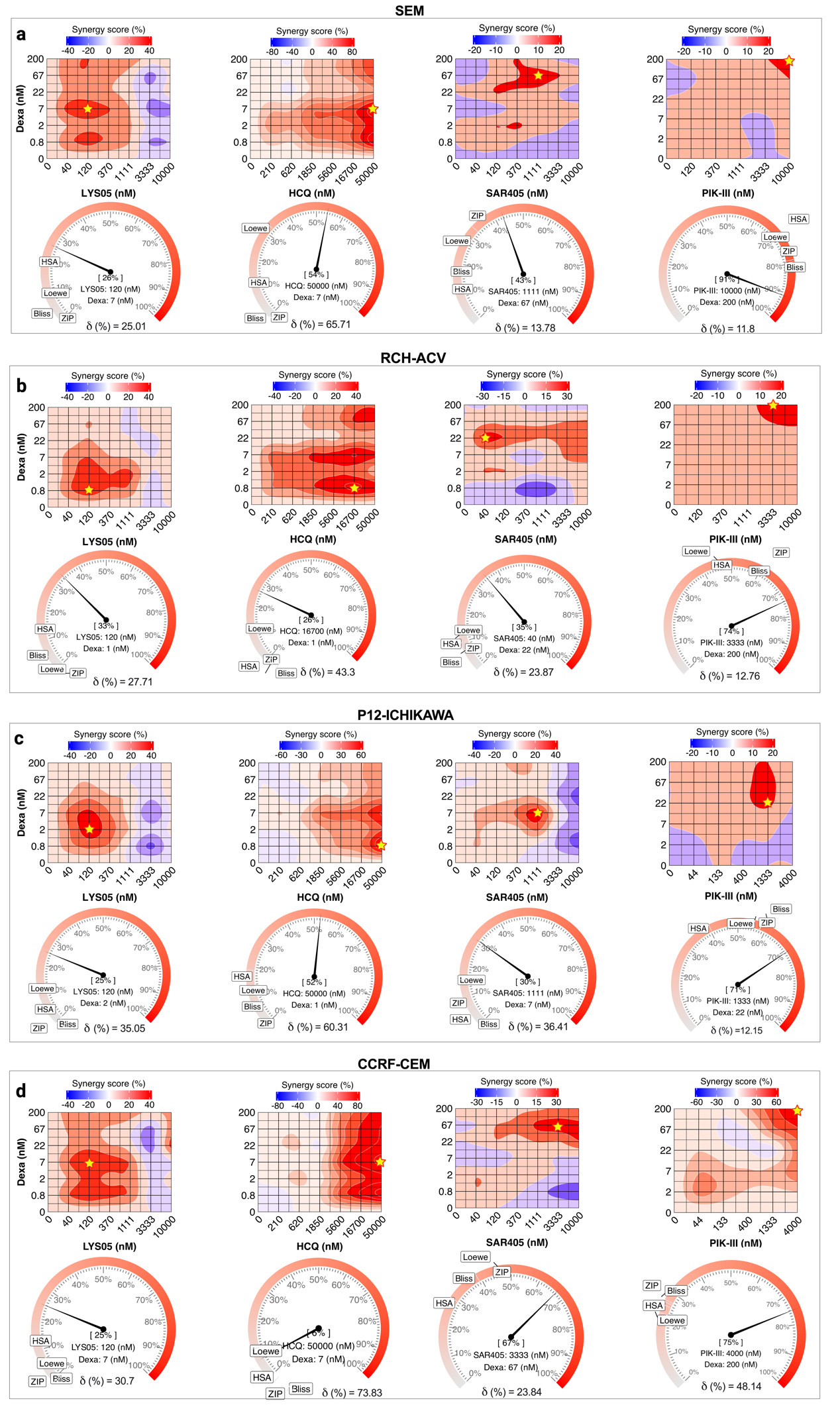


**Supplementary Figure 4: Systematic evaluation of the synergistic effects between dexamethasone and autophagy inhibitors in pre-B- and T-ALL cell lines.** Cells were seeded in an automated manner into 384 well plates containing the indicated dilutions of drugs and their combinations (besides Dexa+PIK-III, which was done manually in 96-well plates), incubated for 72 hours and cell viability was measured using CellTiter-Glo. Data were analyzed using SynergyFinder. **(a-d)** Representative two-drug interaction surface plots for the indicated concentration ranges from two experiments for the indicated cell lines from Figures 3 and 4. Dose combinations with the highest ZIP synergy scores are highlighted with a yellow star in the contour plots **(upper panels)**. The synergy barometer for dose combinations with the highest ZIP (δ%) scores are shown with the δ% values in the **lower panels** for systematic evaluation of synergy at these particular dose combinations. The pointer readout on the barometer indicates the percentage inhibition. The expected percentage inhibition responses of the HSA (Highets Single Agent), LOEWE, BLISS, and ZIP models are indicated on the barometer.


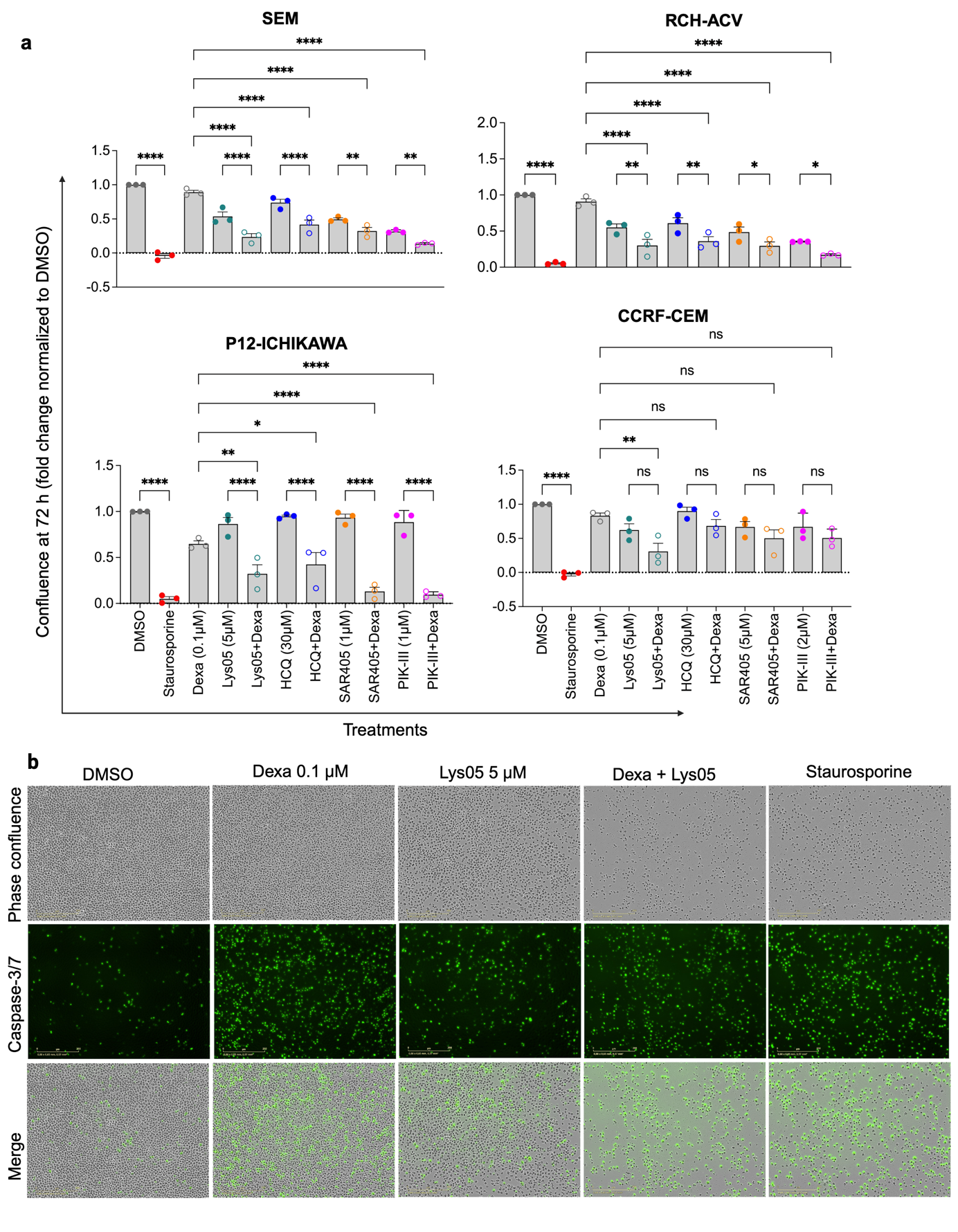


**Supplementary Figure 5: Combination of dexamethasone and autophagy inhibitors show synergistic effects in pre-B- and T-ALL cell lines.** Cells were incubated on Poly-L-orthenine-coated plates for 24 hours and treated with Dexa, indicated autophagy inhibitors or their combinations for 72 hours. CellEvent™ Caspase-3/7 Detection green dye was added to detect cleaved caspase 3/7 in vivo. Cell confluence and cleaved caspase 3/7 fluorescence were monitored using IncuCyte live cell imaging. Treatment with 0.1 µM Staurosporine was used as a positive control for apoptosis induction. **(a)** Bar graphs show the fold change in cell confluence normalized to DMSO at 72 hour time point. Data is representative of mean ±SEM of three independent experiments performed in triplicates **(b)** Representative pictures of CCRF-CEM treated with indicated autophagy inhibitors for 72 hours: phase contrast shows the cell confluence and the green fluorescence shows apoptotic cells with activated Caspase-3/7. * *p* < 0.05; ** *p* < 0.01; *** *p* < 0.001, **** *p* < 0.0001 using one-way ANOVA with Šidák multiple comparison test

**
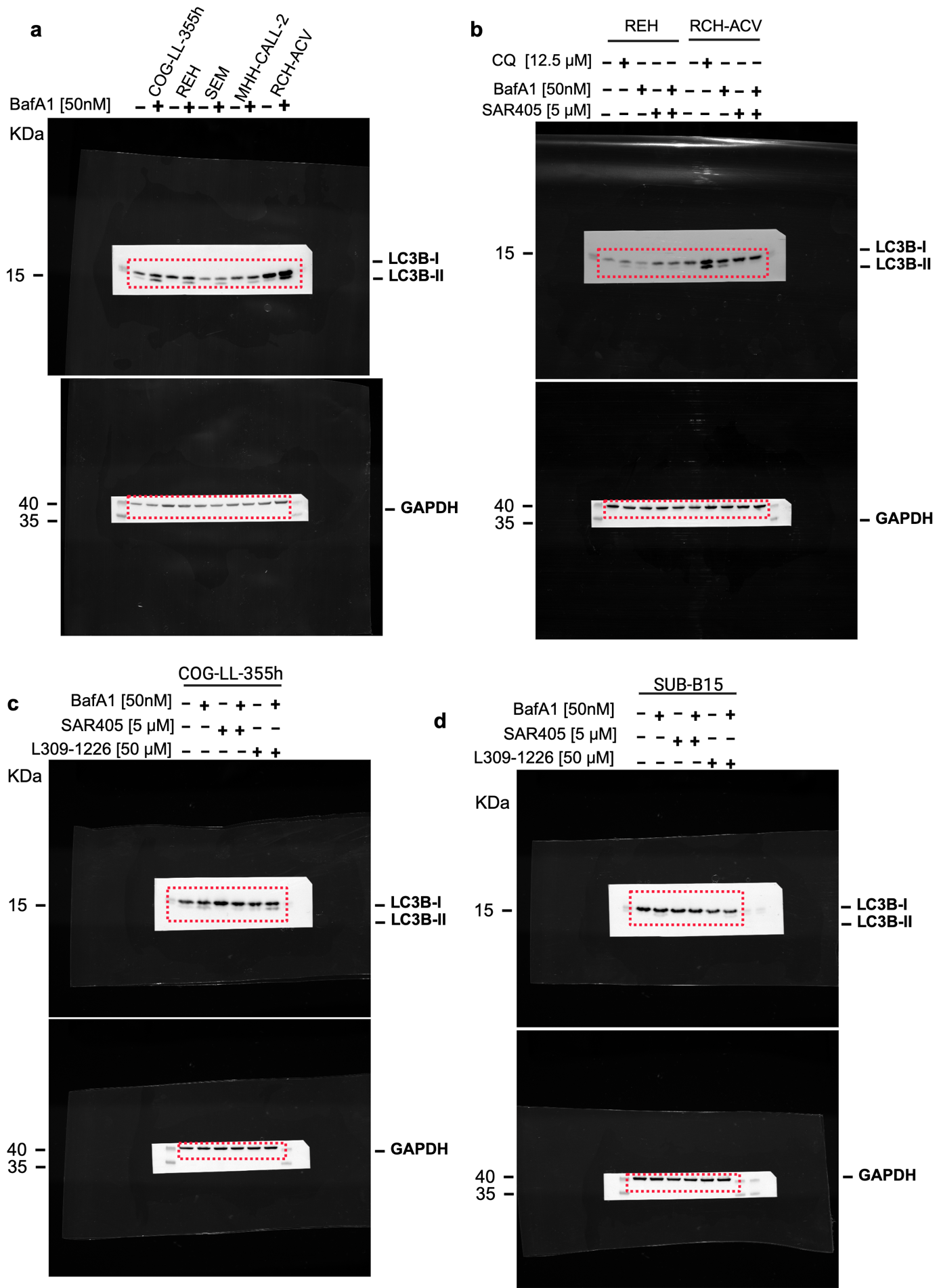
**

**Supplementary Figure 6. Source data uncropped Western blot images from Figures 1e and 2a. The membranes were cut after transfer according to protein size (see Methods) and probed for either LC3B or GAPDH as indicated in the figure. The luminescence and “visible light” images** were recorded using **iBright FL1000/ Invitrogen (ThermoFisher Scientific) and overlaid using iBright FL1000 Imagine system software to visualize the membrane edges. (a)** The chemiluminescence image for Western blotting in Figure 1e overlaid on the “visible light” image of the membrane. **(b)** The chemiluminescence image for Western blotting in the **upper panel** of Figure 2a overlaid on the “visible light” image of the membrane. (**c,d**) The chemiluminescence images for Western blotting in the **lower panels** of Figure 2a, left and right, respectively, overlaid on the “visible light” image of the membrane.

**Supplementary Figure 7. Source data of uncropped Western blots images from Supplementary Figure 3a.**  **The membranes were cut after transfer according to protein size (see Methods) and probed for either GCR or β-actin as indicated in the figure. The visible light images are not available. The exposure time for GAPDH is ten seconds, and therefore edges of the membrane are not visible.**

**SUPPLEMENTARY TABLES**

**Supplementary Table 1**. Leukemia cell line information and medium conditions ^1^

| **Cell Line Name** | **Lineage** | **Tissue** | **Age (Y)** | **Gender** | **Subtype** | **Collection** | **Medium conditions** |
| --- | --- | --- | --- | --- | --- | --- | --- |
| **ALL-SIL** | lymphoblastic_T_cell, T-ALL | BM | 17 | M | NUP214-ABL1 | DSMZ | RPMI.10%FBS |
| **CCRF-CEM** | lymphoblastic_T_cell, T-ALL | PB | 4 | F | T-Other | DSMZ | RPMI.10%FBS |
| **CCRF-HSB2** | lymphoblastic_T_cell, T-ALL | PB | 11,5 | M | T-Other | DSMZ | RPMI.10%FBS |
| **COG-LL-355h** | B-Cell Precursor, pre-B-ALL | BM | - | M | ETV6-RUNX1 | COG | IMDM.20%FBS |
| **KARPAS-45** | lymphoblastic_T_cell, T-ALL | BM | 2 | M | T-Other | ECACC;Sigma | RPMI.10%FBS |
| **KASUMI-9** | B-Cell Precursor, pre-B-ALL | PB | 19 | M | MEF2D-HNRNPUL1 | JCRB | RPMI.10%FBS |
| **LC4-1** | B-Cell Precursor, pre-B-ALL | PB | 13 | F | MEF2D-HNRNPUL1 | JCRB | RPMI.10%FBS |
| **MHH-CALL-2** | B-Cell Precursor, pre-B-ALL | PB | 15 | F | B-Other | DSMZ | RPMI.20%FBS |
| **NALM-6** | B-Cell Precursor, pre-B-ALL | PB | 19 | M | B-Other | DSMZ | RPMI.10%FBS |
| **P12-ICHIKAWA** | lymphoblastic_T_cell, T-ALL | PB | 7 | M | T-Other | DSMZ | RPMI.10%FBS |
| **RCH-ACV** | B-Cell Precursor, pre-B-ALL | BM | 8 | F | TCF3-PBX1 | DSMZ | RPMI.10%FBS |
| **REH** | B-Cell Precursor, pre-B-ALL | PB | 15 | F | ETV6-RUNX1 | ATCC;DSMZ;JCRB | RPMI.10%FBS |
| **SEM** | B-Cell Precursor, pre-B-ALL | PB | 5 | F | KMT2A-AFF1 | DSMZ | RPMI.10%FBS |
| **SUP-B15** | B-Cell Precursor, pre-B-ALL | BM | 9 | M | BCR-ABL1 | ATCC;DSMZ;JCRB | RPMI.10%FBS |
| **697** | B-Cell Precursor, pre-B-ALL | BM | 12 | M | TCF3-PBX1 | DSMZ | RPMI.10%FBS |

**Supplementary Table 2**. DSS2 from the initial drug testing for assessing sensitivity of pre-B-ALL cell lines to a wide range of indicated drugs/inhibitors. Higher DSS values indicate higher sensitivity.

| **Drug/Compound** | **REH** | **COG-LL-355h** | **MHH-CALL-2** | **SUP-B15** | **SEM** | **RCH-ACV** | **KASUMI-9** |
| --- | --- | --- | --- | --- | --- | --- | --- |
| Dexamethasone | 0 | 26,2 | 31,3 | 2 | 9,6 | 15,4 | 0,7 |
| Hydroxychloroquine | 5,2 | 6,6 | 4,6 | 4,4 | 3,3 | 3,2 | 3,3 |
| LYS-01 | 2,6 | 3,7 | 3,4 | 2,9 | 1,8 | 4,8 | 2,1 |
| SAR405 | 3,7 | 5,1 | 6,8 | 2,9 | 6,8 | 6 | 3,4 |

**Supplementary Table 3**. Clinical information of patient-derived primary ALL samples.

| **Sample no.** | **Source tissue** | **ImmunoSubtype** | Translocation/Genotype | Diagnosis/Relapse | % blasts (FACS) | Age at diagnosis/ relapse |
| --- | --- | --- | --- | --- | --- | --- |
| A5085 | Bone marrow | Pre-B-ALL | t(12;21) | 1st relapse | 95 | 4/8 |
| A4333 | Bone marrow | Pre-B-ALL | HeH | Diagnosis | 75 | 5 |
| A4253 | Bone marrow | T-ALL | ND | Diagnosis | 85 | 11 |
| A5126 | Bone marrow | T-ALL | ND | Diagnosis | 90 | 15 |
| A4068 | Bone marrow | Pre-B-ALL | other | Diagnosis | 95 | 8 |
| A2808 | Bone marrow | Pre-B-ALL | (t12;21) | Diagnosis | 93 | 8 |
| A4348 | Bone marrow | Pre-B-ALL | HeH | Diagnosis | 46 | 3 |
| A3834 | Periferal blood | T-ALL | del19p13 (13%) | Diagnosis | 85 | 7 |
| A2315 | Bone marrow | T-ALL | Other | Diagnosis | ? | 10 |
| A2664 | Periferal blood | T-ALL | MLL 11q23 | Diagnosis | 90 | 9 |
| A5272 | Bone marrow | Pre-B-ALL | t(9;22) | 1st Relapse | 64 | 10/16 |

HeH: Hyperdiploid

ND: not defined

**Supplementary Table 4**. Mean Drug Sensitivity Scores, DSS3, of the pre-B-ALL and T-ALL patient samples cultured and treated ex-vivo (n=1).

| **Drug/Compound** | **Pre-B ALL** | | | | | | | **T-ALL** | | | | |
| --- | --- | --- | --- | --- | --- | --- | --- | --- | --- | --- | --- | --- |
|  | **A5085** | **A2808** | **A4068** | **A4079** | **A4348** | **A5272** | **A4333** | **A3834** | **A4253** | **A2315** | **A2664** | **A5126** |
| HCQ | N/A |  | 46.7 | 2.9 | 16.9 | 6.8 | N/A | N/A | 34.8 | 10.9 | 10.2 | N/A |
| Lys05 | 18.9 | 23.8 | 19 | 14.9 | 16 | 11.4 | 12.4 | 35.8 | 5.1 | 7.6 | 20.7 | 6.8 |
| PIK-III | 19 | 11.3 | 1.5 | 0 | 7.9 | 0.1 | N/A | 0 | 0 | 3 | 0 | 1.5 |
| SAR405 | N/A | N/A | 4 | 30.3 | 39 | 4.1 | N/A | N/A | 0.3 | 9.9 | 0.7 | N/A |

1 Leo, I. R. *et al.* Integrative multi-omics and drug response profiling of childhood acute lymphoblastic leukemia cell lines. *Nature communications* **13**, 1691, doi:10.1038/s41467-022-29224-5 (2022).
